# Choroidal neovascularization is inhibited via an intraocular decrease of inflammatory cells in mice lacking complement component C3

**DOI:** 10.1038/srep15702

**Published:** 2015-10-28

**Authors:** Xue Tan, Katsuhito Fujiu, Ichiro Manabe, Junko Nishida, Reiko Yamagishi, Ryozo Nagai, Yasuo Yanagi

**Affiliations:** 1 Department of Ophthalmology, The University of Tokyo, Tokyo, Japan; 2 Department of Cardiovascular Medicine, Graduate School of Medicine and Faculty of Medicine, The University of Tokyo, Tokyo, Japan; 3 Department of Ubiquitous Health Informatics, School of Medicine, The University of Tokyo, Tokyo, Japan; 4 Precursory Research for Embryonic Science and Technology, Japan Science and Technology Agency, Tokyo, Japan; 5 Jichi Medical University, Tochigi, Japan; 6 Singapore Eye Research Institute, Singapore, Singapore; 7 Medical Retina Department, Singapore National Eye Center, Singapore, Singapore

## Abstract

In early age-related macular degeneration (AMD), complement component C3 can be observed in drusen, which is the accumulation of material beneath the retinal pigment epithelium. The complement pathways, via the activation of C3, can upregulate the expression of cytokines and their receptors and the recruitment of inflammatory leukocytes, both of which play an important role in the development of choroidal neovascularization (CNV) in exudative AMD. Laser-induced CNV lesions were found to be significantly smaller in *C3*^*−/−*^ mice than in wild-type mice. By using flow cytometry, we demonstrated that the proportions of intraocular granulocytes, CD11b^+^F4/80^+^Ly6C^hi^ and CD11b^+^F4/80^+^Ly6C^lo^ cells, were lower in *C3*^*−/−*^ mice than in wild-type mice as early as day 1 after laser injury, and the proportions of granulocytes and three macrophage/monocyte subsets were significantly lower on day 3. In contrast, *C3*^*−/−*^ mice had more granulocytes and CD11b^+^F4/80^+^Ly6C^hi^ cells in peripheral blood than wild-type mice after injury. Further, the expression levels of *Vegfa164* were upregulated in intraocular Ly6C^hi^ macrophages/monocytes of *C3*^*−/−*^ mice, but not as much as in wild-type mice. Collectively, our data demonstrate that despite a more pronounced induction of systemic inflammation, inhibition of complement factor C3 suppresses CNV by decreasing the recruitment of inflammatory cells to the lesion.

Age-related macular degeneration (AMD) is among the most common causes of blindness in developed countries^1^. Exudative AMD is characterized by the growth of abnormal blood vessels, called choroidal neovascularization (CNV). Leakage from these vessels under or into the retina is the major cause of vision loss^2^, and these changes usually occur after lipofuscin has accumulated between the retinal pigment epithelium (RPE) and Bruch’s membrane^3^. Lipofuscin accumulation is observed in healthy as well as diseased eyes, but it has been demonstrated that abnormal lipofuscin accumulation is related to drusen formation^4^.

Previous studies have demonstrated that drusen are the product of local inflammation resulting from RPE disorders involving the immune system^3^,^5^, and the complement component C3 is contained in the drusen of patients with AMD^6^. The alternative pathway is activated by spontaneous hydrolysis of C3. The anaphylatoxins produced by C3 hydrolysis, such as C3a and C3b, and their downstream factors, including C5a, are important chemoattractants that can trigger the recruitment of neutrophils and macrophages^7^,^8^. In a mouse CNV model, both C3a and C5a have been suggested to contribute to angiogenesis and upregulate the expression of vascular endothelial growth factor (VEGF)^9^. The alternative pathway via C3b anaphylatoxin can also lead to the formation of the membrane attack complex (MAC) C5b-9^8^. It has been demonstrated that inhibition of MAC formation results in the inhibition of CNV in mice^10^.

The involvement of inflammatory cells, including macrophages, in the pathogenesis of exudative AMD has been reported in histologic studies of CNV^11^,^12^. In experimental models, macrophages and granulocytes have also been found to infiltrate laser-induced CNV lesions^13^,^14^. Many studies have suggested that macrophage depletion correlates with reduced CNV responses^14^,^15^, although others have demonstrated that an intraocular injection of macrophages reduces CNV size^16^,^17^. These contradictory findings suggest that the effect of macrophages on CNV is complex and may depend on the age of the mice used and the age and subtypes of macrophages.

The bone marrow and peripheral blood are the primary sources from which monocytes are mobilized to enter into tissue sites after injury^18^. There are three subsets of macrophages (Ly6C^hi^, Ly6C^int^, and Ly6C^lo^) that are derived from circulating inflammatory monocytes in mouse models of chronic disease^19^. Ly6C^hi^ macrophages/monocytes, which are similar to M1 macrophages, migrate to injured tissues and produce pro-inflammatory cytokines and chemokines in a mouse model of arteriosclerosis, chronic heart failure, and chronic kidney failure^20^,^21^,^22^. In contrast, Ly6C^lo^ macrophages/monocytes that differentiate into the M2 macrophage subtype promote wound healing in myocardium, inflamed skeletal muscle, and brain tissues^23^,^24^,^25^. Ly6C^hi^ monocytes have been demonstrated to leave the bone marrow and enter the circulating blood via CC-chemokine receptor 2-mediated migration^26^. In the steady state, Ly6C^hi^ macrophages/monocytes differentiate into Ly6C^lo^ macrophages/monocytes in the circulation^27^. Ly6C^lo^ macrophages/monocytes are recruited into tissue via the chemokine receptor CX3CR1^28^ and are considered to become tissue-resident macrophages; however, recent studies suggest tissue-resident macrophages originate from the yolk sac or fetal liver progenitors and self-renew *in situ*^29^,^30^,^31^. Ly6C^int^ macrophages/monocytes are thought to be a phenotype that the Ly6C^hi^ subtype adopts (under steady-state conditions) before they form the Ly6C^lo^ subtype^32^. Moreover, their functions depend on the context in which they are found^32^,^33^.

To test directly the involvement of C3 in the recruitment of inflammatory cells in the development of CNV, we generated laser-induced CNV in wild-type and C3-deficient (*C3*^*−/−*^) mice. First, we compared lesion size between wild-type and *C3*^*−/−*^ mice. Second, we used flow cytometry to evaluate the time course of changes in the proportions of granulocytes and macrophage subsets in the posterior segment of the eye and the peripheral blood in both types of mice after laser photocoagulation. Lastly, we examined the expression of *Vegfa164* and *Vegfr1* in intraocular Ly6C^hi^ macrophages/monocytes with or without laser treatment.

## Results

We used *C3*^*−/−*^ mice and their wild-type littermates to investigate the role of C3 in CNV. *C3*^*−/−*^ mice and wild-type mice were all *Rd1* and *Rd8* negative and had similar retinal morphology in the absence of laser treatment. At the age of 6 months, no drusen, retinal lesions, or immune cell infiltration were detected in mice of either genotype (data not shown).

**Figure 1:**
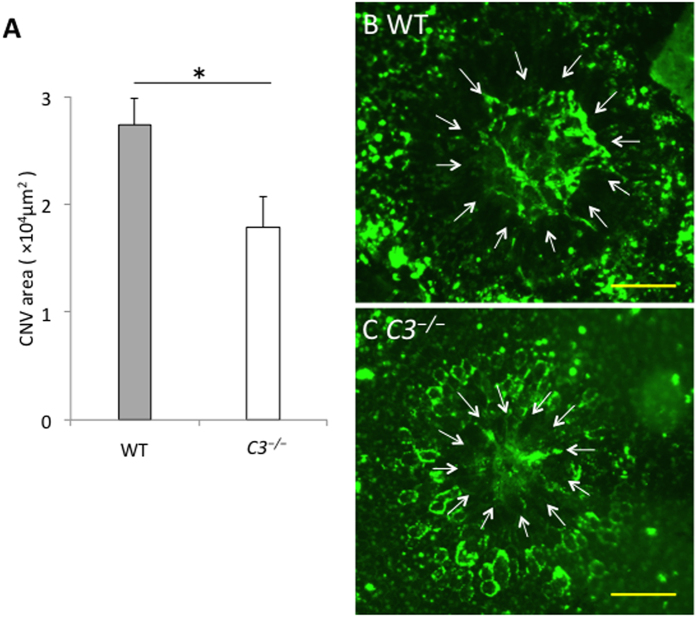
Lesion size after laser photocoagulation. At 7 days after laser injury, the mice were perfused transcardially with PBS and then with FITC-conjugated concanavalin A (20 μg/mL in PBS) to label the choroidal neovascularization (CNVs). The eyes were removed and retinal pigment epithelium (RPE)-choroid flatmounts were prepared. Measurements of CNV lesion area were carried out using ImageJ. The outline of the CNV was drawn around the contour of the lesion and then the total lesion area was measured. The average area obtained from 4 lesions in each eye was used for analysis. (**A**) Lesion size was significantly smaller in *C3*^*−/−*^ mice than in wild-type (WT) mice. (**B**) The CNV in a representative WT mouse (white arrows). (**C**) The CNV lesion in a representative *C3*^*−/−*^ mouse is smaller than that in the WT mouse shown in B. Scale bars, 100 μm. *P < 0.05 compared with WT mice. n = 6 for each group.

Male mice 7 to 8 weeks of age were used for the laser-induced CNV mouse model. At 7 days after laser injury, *C3*^*−/−*^ mice had lesions that were 35% smaller than those in wild-type mice (*P* < 0.05; Fig. 1A–C).

**Figure 2:**
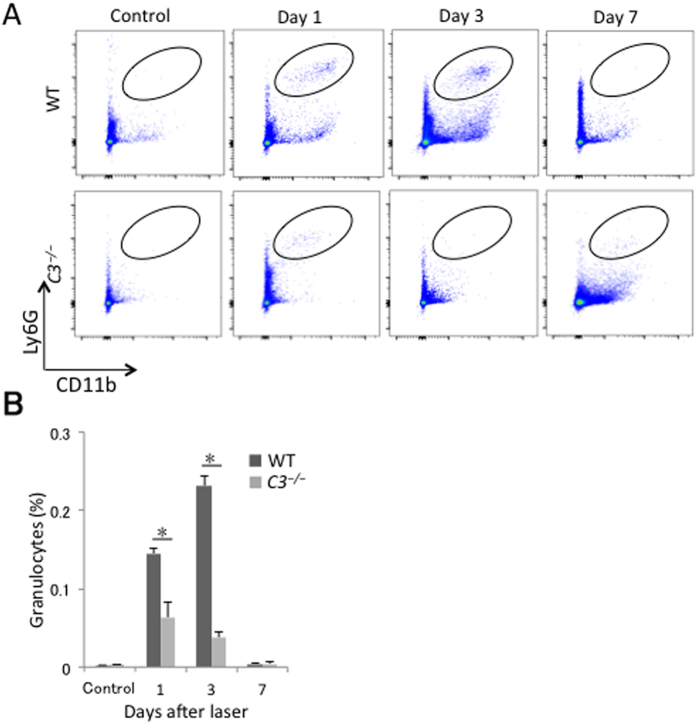
Time course of changes in the proportion of granulocytes recruited into the posterior segment of the eye in response to laser photocoagulation. (**A**) Representative flow cytometry plots of granulocytes in the posterior segment of the eye from WT and *C3*^*−/−*^ mice without laser treatment and at 1, 3, and 7 days after injury. The regions surrounded by ovals contain CD11b^+^Ly6G^+^ cells. (**B**) Ratios of CD11b^+^Ly6G^+^ cells (oval region in A) to total number of live cells. *P < 0.05 compared with WT mice. Six mice were used to give a single value; n = 3 in each experiment.

In wild-type mice, the proportion of granulocytes recruited into the posterior segment of the eye among the total number of cells increased greatly from day 1 after laser injury and peaked on day 3. In contrast, the increase in the proportion of granulocytes in *C3*^*−/−*^ mice was moderate; it peaked at 1 day after laser injury and decreased thereafter, and was significantly lower on days 1 (43.8 ± 11.0%) and 3 (16.5 ± 3.9%) than in wild-type mice (Fig. 2A,B).

**Figure 3:**
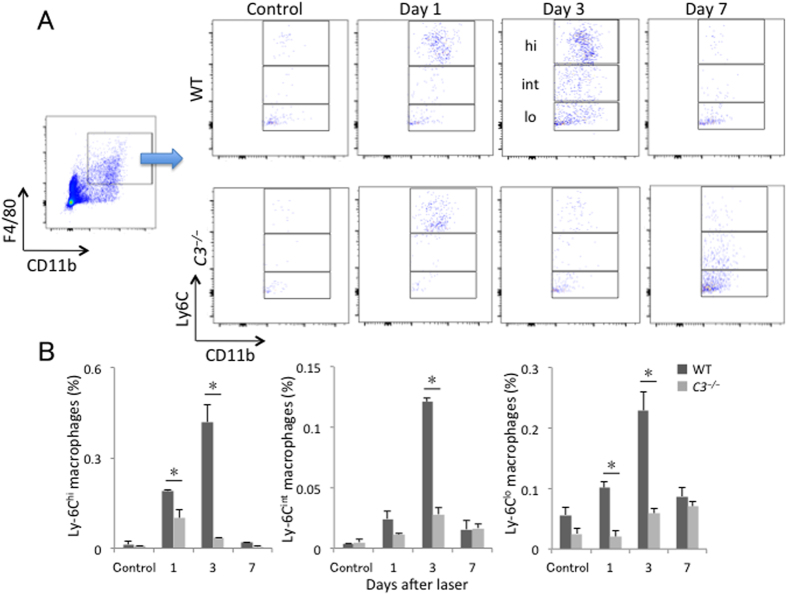
Lack of C3 affected the recruitment of macrophage/monocyte subsets to the posterior segment of the eye. (**A**) Representative flow cytometry plots of macrophages/monocytes in the posterior segment of the eye from WT and *C3*^*−/−*^ mice without laser treatment and at 1, 3, and 7 days after injury. Hi, int, and lo correspond to CD11b^+^F4/80^+^Ly6C^hi^, CD11b^+^F4/80^+^Ly6C^int^, and CD11b^+^F4/80^+^Ly6C^lo^ cells, respectively. (**B**) Ratios of CD11b^+^F4/80^+^Ly6C^hi^ (region hi in **A**), CD11b^+^F4/80^+^Ly6C^int^ (region int in **A**), and CD11b^+^F4/80^+^Ly6C^lo^ (region lo in **A**) cells to total number of live cells. *P < 0.05 compared with the same subtype of WT mice. Six mice were used to give a single value; n = 3 in each experiment.

The three subsets of intraocular macrophages/monocytes (Ly6C^hi^, Ly6C^int^, and Ly6C^lo^) peaked at 3 days after laser injury in wild-type mice. Compared with wild-type mice, the increase in Ly6C^hi^ and Ly6C^lo^ macrophages/monocytes was suppressed from day 1 after injury in *C3*^*−/−*^ mice (to 52.0 ± 14.9% and 20.5 ± 11.5% of wild-type values, respectively), and the three macrophage/monocyte subsets were significantly lower on day 3 after injury relative to those in wild-type mice (8.0 ± 1.3%, 23.1 ± 4.1%, and 25.5 ± 0.7% of wild-type values, for Ly6C^hi^, Ly6C^int^, and Ly6C^lo^, respectively; *P* < 0.05 for all comparisons) (Fig. 3A,B).

**Figure 4:**
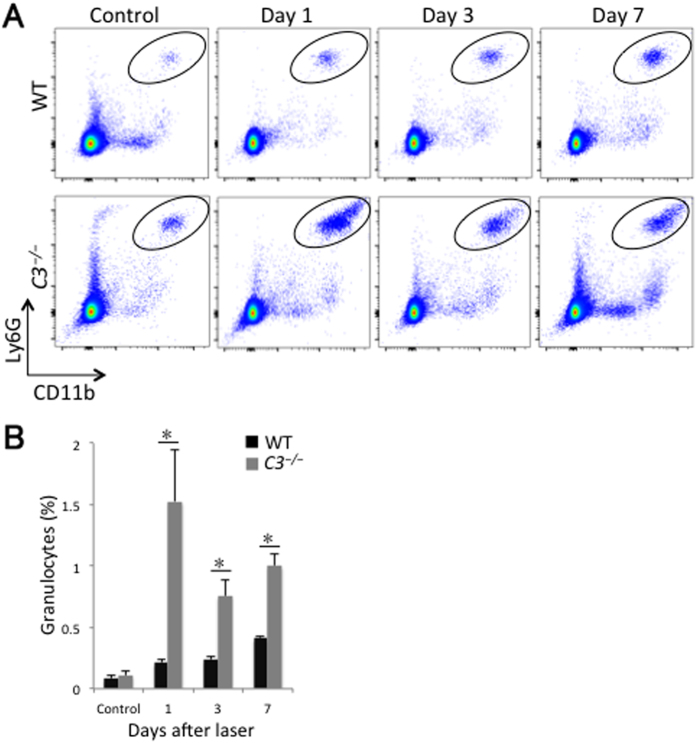
Change in the percentage of granulocytes circulating in peripheral blood after laser injury. (**A**) Representative flow cytometry plots of CD11b^+^Ly6G^+^ cells (in ovals) in peripheral blood from WT and *C3*^*−/−*^ mice. There was no significant difference in the number of granulocytes between WT and *C3*^*−/−*^ mice without treatment. Although the proportion of granulocytes circulating in peripheral blood in WT mice increased until day 7 after laser treatment, significantly higher proportions of granulocytes were observed in *C3*^*−/−*^ mice than in WT mice at 1, 3, and 7 days after laser injury. (**B**) Ratios of CD11b^+^Ly6G^+^ cells (oval region in **A**) to total number of live cells. *P < 0.05 compared with WT mice. Six mice were used to give a single value; n = 3 in each experiment.

**Figure 5:**
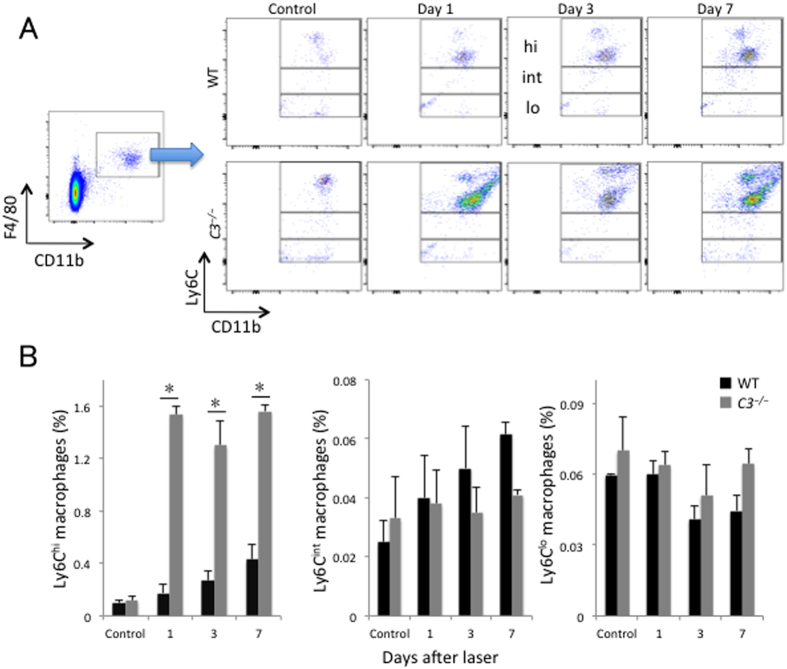
Change in the percentage of macrophage/monocyte subsets circulating in peripheral blood after laser injury. (**A**) Representative flow cytometry plots of CD11b^+^F4/80^+^Ly6C^hi^, CD11b^+^F4/80^+^Ly6C^int^, and CD11b^+^F4/80^+^Ly6C^lo^ cells in peripheral blood from WT and *C3*^*−/−*^ mice. A no-stain control was used to exclude cells with spontaneous fluorescence. There was no significant difference in the proportions of macrophages/monocytes between WT and *C3*^*−/−*^ mice without treatment. Although CD11b^+^F4/80^+^Ly6C^hi^ cells circulating in peripheral blood in WT mice increased until day 7 after laser treatment, significantly higher proportions of CD11b^+^F4/80^+^Ly6C^hi^ cells were observed in *C3*^*−/−*^ mice than in WT mice at 1, 3, and 7 days after laser injury. There was no significant difference in the proportions of CD11b^+^F4/80^+^Ly6C^lo^ and CD11b^+^F4/80^+^Ly6C^int^ cells in blood between both groups during CNV formation. Hi, int, and lo correspond to CD11b^+^F4/80^+^Ly6C^hi^, CD11b^+^F4/80^+^Ly6C^int^, and CD11b^+^F4/80^+^Ly6C^lo^ cells, respectively. (**B**) Ratios of CD11b^+^F4/80^+^Ly6C^hi^ (region hi in A), CD11b^+^F4/80^+^Ly6C^int^ (region int in A), and CD11b^+^F4/80^+^Ly6C^lo^ (region lo in A) cells to total number of live cells. *P < 0.05 compared with the same subtype of WT mice. Six mice were used to give a single value; n = 3 in each experiment.

Considering that circulating inflammatory cells migrate into the lesion, we next asked whether there were any changes in the inflammatory cells circulating in peripheral blood. There was no significant difference in the number of granulocytes and macrophages/monocytes between wild-type and *C3*^*−/−*^ mice without treatment (Figs 4B and 5B). Although granulocytes and Ly6C^hi^ macrophages/monocytes circulating in peripheral blood in wild-type mice subsequently increased until day 7 after laser treatment, significantly higher percentages of granulocytes were observed in *C3*^*−/−*^ mice than in wild-type mice at days 1 (7.3 ± 2.0-fold compared with wild-type values), 3 (3.2 ± 0.6-fold compared with wild-type values), and 7 after laser injury (2.4 ± 0.2-fold compared with wild-type values; *P* < 0.05 for all comparisons) (Fig. 4B). With regard to Ly6C^hi^ macrophages/monocytes, *C3*^*−/−*^ mice also had more Ly6C^hi^ cells in the peripheral blood than wild-type mice at days 1 (8.9 ± 0.3-fold compared with wild-type values), 3 (4.9 ± 0.7-fold compared with wild-type values), and 7 after laser injury (3.6 ± 0.1-fold compared with wild-type values; *P* < 0.05 for all comparisons) (Fig. 5B). There was no significant difference in the proportions of Ly6C^lo^ and Ly6C^int^ macrophages/monocytes in the blood between the two groups during CNV formation (Fig. 5). Moreover, there was no significant change in the percentages of splenic and myeloid inflammatory cells between both groups (data not shown).

**Figure 6:**
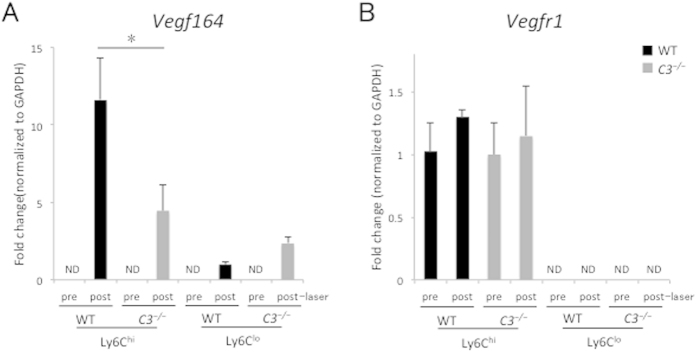
Fold changes in *Vegfa164* and *Vegfr1* expression levels before and after laser. Ly6C^hi^ and Ly6C^lo^ cells were sorted from the eyes of WT and *C3*^*−/−*^ mice without or with laser treatment (3 days after laser injury) by using flow cytometry. Real-time RT-PCR was performed. *Vegfa164* expression was not detected in intraocular Ly6C^hi^ or Ly6C^lo^ macrophages/monocytes in both groups without laser treatment. In contrast, *Vegfa164* expression levels were upregulated in intraocular Ly6C^hi^ cells of *C3*^*−/−*^ mice after laser injury, but not as much as in those of WT mice. There was no significant difference in the expression levels of *Vegfr1* in intraocular Ly6C^hi^ cells between both groups before or after laser injury. *Vegfr1* expression was not detected in intraocular Ly6C^lo^ cells of both groups with or without laser treatment. *P < 0.05 compared with WT mice. Ten mice were used to give a single value; n = 3 in each experiment.

This seemingly discrepant increase in inflammatory cells in the blood and decrease in cells infiltrating the eye led us to investigate *Vegf* and one of its receptors, *Vegfr1*, which is expressed on monocytes and functions as a chemoattractant receptor. To investigate *Vegf* and *Vegfr1* expression in intraocular inflammatory cells in both groups before and after laser injury, we performed real-time quantitative reverse transcriptase-polymerase chain reaction (RT-PCR). *Vegfa164* expression was barely detected in intraocular Ly6C^hi^ or Ly6C^lo^ macrophages/monocytes in both groups without laser treatment. In contrast, *Vegfa164* expression levels were upregulated in intraocular Ly6C^hi^ cells after laser injury; however, the upregulated expression in *C3*^*−/−*^ mice (4.5 ± 1.7-fold compared with the Ly6C^lo^ values of wild-type mice) was significantly lower than in wild-type mice (11.6 ± 2.7-fold compared with the Ly6C^lo^ values of wild-type mice) (Fig. 6A). There was no significant difference in the expression levels of *Vegfr1* in intraocular Ly6C^hi^ cells between both groups before or after laser injury. *Vegfr1* expression was not detected in intraocular Ly6C^lo^ cells of both groups with or without laser treatment (Fig. 6B).

## Discussion

Prior studies have demonstrated that the activation of complement component C3 in drusen can induce chronic inflammation in the RPE and play an important role in angiogenesis in AMD^3^,^5^,^6^. Recent studies have also described the importance of C3 and the C3a receptor in leukocyte recruitment to the choroid after laser treatment in a laser-induced CNV model^9^,^34^. In contrast, another group reported that *C3*^*–/–*^ mice have increased neovascularization^35^. In this study, we investigated the effects of C3 on CNV lesion size and the involvement of C3 deficiency in inflammatory cell recruitment, such as granulocytes and macrophage/monocyte subsets, during the formation of CNV.

The result that CNV lesion size was significantly decreased in *C3*^*−/−*^ mice was consistent with a previous report^34^, and several groups have reported that maximal neutrophil infiltration into the choroid occurs 1 day after laser injury^9^,^36^. However, others have reported that neutrophil infiltration is maximal at 3 days after laser injury in wild-type mice^13^. All studies concur that macrophage infiltration peaks on day 3 after laser injury^9^,^36^,^37^. In our study, granulocytes and all three macrophage subsets showed maximal infiltration into the posterior segment of the eyes of wild-type mice 3 days after laser treatment. However, in *C3*^*−/−*^ mice, the proportions of intraocular granulocytes and Ly6C^hi^ macrophages were maximal at 1 day after laser injury, whereas Ly6C^int^ macrophages were maximal at 3 days and the proportion of Ly6C^lo^ macrophages increased until at least 7 days. In sharp contrast to the larger proportion of inflammatory cells in the peripheral blood of *C3*^*−/−*^ mice, immune cell infiltration of the eyes was markedly suppressed by C3 deficiency. It is likely that tissue migration and the recruitment of immune cells were inhibited because of the lack of C3a and C5a anaphylatoxins. It is also plausible that when the number of inflammatory cells infiltrating the eyes is increased, a feedback loop may occur and reduce the number of inflammatory cells in the peripheral blood^38^. Our results also suggest that this feedback mechanism might be abrogated in *C3*^*−/−*^ mice, probably due to the deletion of C3 and subsequent loss of anaphylatoxin production.

Complement C3 is produced in the RPE/choroid and upregulated in the CNV mouse model^34^,^39^. C3a and C5a anaphylatoxins have been shown to regulate tissue infiltration of macrophages and monocytes, and control VEGF production and secretion from RPE cells. RPE cells also contribute to the VEGF-VEGFR1 axis. It is likely that macrophage/monocyte recruitment into the eye and the upregulation of VEGF expression are suppressed in *C3*^*−/−*^ mice because of the absence of C3a and C5a. Moreover, the inhibition of laser-induced CNV in *C3*^*−/−*^ mice is likely to be due to the reduction of VEGF expression in the eye. However, further studies are still required to identify the role of C3 in the production of VEGF by RPE cells and to clarify whether locally or systematically produced C3 plays a pivotal role.

Blockade of the complement pathway is a new strategy for the treatment of AMD. Many new drugs are currently being studied in clinical trials. POT-4 (Potentia Pharmaceuticals, Louisville, KY) is a peptide capable of binding to human complement factor C3 and preventing its activation, and a phase I clinical trial of POT-4 for exudative AMD has been completed^40^. Our findings provide additional evidence that an ocular C3-targeting agent is likely to be effective in the treatment of exudative AMD.

In conclusion, our data show that inhibition of intraocular complement factor C3 might suppress CNV formation via reduction of macrophage/granulocyte infiltration and *Vegfa164* expression. Thus, a C3 inhibitor might be useful in the treatment of AMD. Importantly, our results also support the pro-angiogenic nature of Ly6C^hi^ macrophages/monocytes in CNV.

## Methods

### Ethics statement

All animal experiments were performed in accordance with the guidelines of the University of Tokyo and the Association for Research in Vision and Ophthalmology. All experimental protocols were approved by the department’s animal experimentation committee of the University of Tokyo.

### Animals

Male wild-type C57BL/6J mice were purchased from Kiwa Laboratory (Wakayama, Japan). Male C3-deficient mice (*C3*^*−/−*^), backcrossed into a C57BL/6 background, were kindly provided by Dr. Naito (Osaka University Graduate School of Medicine). The mice were used at 7 to 8 weeks of age. Genotyping was performed as described previously and also to exclude the presence of *Rd1* and *Rd8* mutations^41^,^42^,^43^. Anesthesia was achieved by intramuscular injection of 75 mg/kg ketamine HCL (Ketalar^®^; Sankyo, Tokyo, Japan) and 5 mg/kg xylazine (Celectal^®^; Bayer, Tokyo, Japan). Pupils were dilated with a solution of 0.5% tropicamide (Mydrin-M^®^; Santen, Osaka, Japan).

### Laser-induced CNV

CNV lesions were induced by laser photocoagulation. Laser radiation was delivered between the major retinal vessels and at equal distances from the optic disc with a diode laser (DC-3000^®^; NIDEK, Osaka, Japan) and a slit lamp delivery system (SL-7F; Topcon, Tokyo, Japan). Through a glass cover used as a contact lens, we delivered laser light (200 mW intensity, 170 μm size, 0.02 s duration) to 4 spots per eye for the lesion size study and to 12 spots per eye for the flow cytometry studies. The rupture of Bruch’s membrane for each lesion was evidenced by bubble formation at the time of laser exposure^44^,^45^. Each experimental group consisted of 6 or 10 mice. For the analysis of CNV area, 6 mice per group were subjected to laser treatment and used for the analysis. For flow cytometric analysis, cells isolated from 6 mice were used for a single FACS analysis and cells from 10 mice were used for a single RNA isolation protocol. The results from three independent experiments are shown. Thus, a total of 54 wild-type mice and 54 *C3*^*−/−*^ mice were used for the experiments.

### RPE flatmounts

At 7 days after laser injury, the mice were perfused transcardially with phosphate-buffered saline (PBS) and then with FITC-conjugated concanavalin A (20 μg/mL in PBS; Vector Laboratories, Burlingame, CA) to label the CNVs. The eyes were removed and the posterior segment of the eyeball was prepared as a flatmount. RPE flatmounts were observed using a fluorescence microscope (Keyence Corporation, Tokyo, Japan). Measurements of CNV lesion area were performed using ImageJ software (developed by Wayne Rasband, National Institutes of Health, Bethesda, MD; available at http://rsbweb.nih.gov/ij/index.html). The outline of the CNV was drawn around the perimeter of the lesion and then total lesion area was measured^46^. The average CNV area obtained from 4 lesions in each eye was used to give a single value for analysis.

### Flow cytometry

To isolate cells from eyes, retinas and RPE/choroids were harvested from 12 eyes (6 mice) of each group and combined in collagenase (1 mg/mL; Wako, Osaka, Japan) and dispase (1 mg/mL; Invitrogen, Waltham, MA, USA) in PBS. After 10 min incubation at 37 °C, the cells were passed through a 40-μm nylon mesh and then centrifuged at 700 × *g* for 5 min at 4 °C. The supernatant was discarded, and cells resuspended with PBS were kept on ice for flow cytometry. Peripheral blood was collected from the heart with 1000 U/mL heparin (Novo-Heparin^®^; Mochida, Tokyo, Japan). After the spleens and femurs were harvested, the cells were isolated from them as described previously^47^. After single cell suspensions were prepared as described previously^48^, the samples were subjected to flow cytometric analyses. All analyses were performed using a FACSAria III (BD Biosciences, San Jose, CA, USA) and FlowJo software (Tree Star, Ashland, OR, USA). Anti-F4/80-APC, anti-Ly6C-FITC, and anti-Ly6G-APC-Cy7 (all from BioLegend, San Diego, CA, USA) and anti-CD11b-PE (eBioscience, San Diego, CA, USA) were used for the flow cytometric analyses. Granulocytes and macrophage/monocyte subsets were identified as CD11b^hi^F4/80^lo^Ly6G^hi^ and CD11b^hi^F4/80^hi^Ly6C^hi/int/lo^, respectively. Changes of inflammatory cells over time were evaluated without laser treatment and at days 1, 3, and 7 after laser injury. Ly6C^hi^ and Ly6C^lo^ cells were sorted from the retina and RPE/choroids of wild-type and *C3*^*−/−*^ mice without laser treatment or at 3 days after injury by using the FACSAria III (BD Biosciences)^48^.

### Real-time RT-PCR

RNA from macrophages sorted by fluorescence-activated cell sorting was extracted using an RNeasy Mini Kit (QIAGEN, Valencia, CA, USA), and RT was performed using SuperScript® VILO™ Master Mix (Invitrogen) in accordance with the manufacturer’s protocol. Real-time RT-PCR was performed using Platinum SYBR Green qPCR SuperMix UDG (Invitrogen) in a Thermal Cycler Dice^®^ Real Time System II (Takara, Shiga, Japan) in accordance with the manufacturer’s protocol. *Vegfa164* and *Vegfr1* expression levels in intraocular Ly6C^hi^ and Ly6C^lo^ cells of wild-type and *C3*^*−/−*^ mice were evaluated. Values were normalized to the expression levels of *Gapdh*. The primer sequences for mouse *Vegfa164* were forward, 5′-GCCAGCACATAGGAGAGATGAGC-3′; and reverse, 5′-CAAGGCTCACAGTGATTTTCTGG-3′. The primer sequences for mouse *Vegfr1* were forward, 5′-GAGGAGGATGAGGGTGTCTATAGGT-3′; and reverse, 5′-GTGATCAGCTCCAGGTTTGACTT-3′. The primer sequences for mouse *Gapdh* were forward, 5′-CACATTGGGGGTAGGAACAC-3′; and reverse, 5′-AACTTTGGCATTGTGGAAGG-3′.

### Statistical analysis

Unpaired *t* tests were performed to compare the differences between the groups. *P* values less than 0.05 were considered statistically significant.

## Additional Information

**How to cite this article**: Tan, X. *et al* Choroidal neovascularization is inhibited via an intraocular decrease of inflammatory cells in mice lacking complement component C3. *Sci. Rep.* **5**, 15702; doi: 10.1038/srep15702 (2015).
